# 组蛋白去乙酰化酶抑制剂联合免疫检查点抑制剂治疗肿瘤的研究进展

**DOI:** 10.3779/j.issn.1009-3419.2021.102.11

**Published:** 2021-03-20

**Authors:** 晨 方, 勇 王, 勇 李

**Affiliations:** 330000 南昌，南昌大学第一附属医院肿瘤科 Department of Medical Oncology, The First Affiliated Hospital of Nanchang University, Nanchang 330000, China

**Keywords:** 组蛋白去乙酰化酶抑制剂, 免疫检查点抑制剂, 免疫耐药, 免疫应答, 恶性肿瘤, Histone deacetylase inhibitor, Immune checkpoint inhibitors, Immunotherapy resistance, Immune response, Cancer

## Abstract

免疫治疗是目前抗肿瘤治疗的主要策略之一，其中免疫检查点抑制剂（immune checkpoint inhibitors, ICIs）是研究应用最为广泛的一类药物。ICIs耐药由多种细胞因子及免疫细胞介导，机制复杂，是造成癌症患者免疫治疗失败的主要原因。组蛋白去乙酰化酶抑制剂（histone deacetylase inhibitor, HDACi）作为一类表观遗传调控药物，在调控细胞周期、增殖分化及活性中发挥重要作用。近年来，研究发现HDACi不仅能够调节细胞生物学特征，还与肿瘤ICIs耐药的改善密切相关。因此，HDACi提高ICIs疗效的相关机制研究对肿瘤免疫治疗具有重要意义。本文将对HDACi联合ICIs治疗恶性肿瘤及相关机制的研究进展做一综述。

## Abstract (en)

Immunotherapy is one of the main strategies of anti-tumor therapy at present, in which immune checkpoint inhibitors (ICIs) are the most widely used drug. ICIs resistance is mediated by a variety of cytokines and immune cells, and the mechanism is complex, which is the main reason for the failure of immunotherapy in cancer patients. Histone deacetylase inhibitor (HDACi), as a class of epigenetic regulatory drugs, plays an important role in regulating cell cycle, proliferation, differentiation, and activity. In recent years, Studies have found that HDACi can not only regulate cell biological characteristics, but also closely related to the improvement of tumor ICIs drug resistance. Therefore, the study on how HDACi enhances the efficacy of ICIs is of great significance to tumor immunotherapy. This article will review the research progress of HDACi combined with ICIs in treating malignant tumors and their related mechanism.

恶性肿瘤是当前威胁人类健康的一类重大疾病，近年来肿瘤免疫治疗受到广泛关注及深入研究，其中免疫检查点抑制剂（immune checkpoint inhibitors, ICIs）在多个肿瘤治疗领域取得突破性进展，为患者带来新的治疗希望^[1, 2]^。然而，相当部分患者对ICIs无应答、产生耐药或用药后病情出现新进展的情况不容忽视^[3-5]^，研究^[6]^表明，ICIs联合表观遗传调控药物组蛋白去乙酰化酶抑制剂（histone deacetylase inhibitor, HDACi）可显著逆转ICIs耐药现象并放大免疫治疗效果，本文主要就HDACi改善ICIs耐药的相关机制进行阐述。

## 1 HDACi

大量研究^[7]^发现，多种类型癌症中异常表达的组蛋白去乙酰化酶（histone deacetylase, HDAC）能通过诱导细胞增殖、细胞迁移、血管生成和抑制凋亡等生物学途径促进肿瘤发生，而相应HDACi的研发使用为肿瘤精准治疗提供新的思路^[8]^。HDACi作为生物类抑制剂，在癌症治疗中用作表观遗传调控药物，通过干扰HDAC的活性间接诱导组蛋白乙酰化来调节基因表达。多项研究^[8-10]^显示，HDACi可以使受抑制的调节基因在癌细胞中重新表达并逆转其恶性表型。并且，通过调节生物活动过程，如诱导细胞周期阻滞、抑制血管生成、调节免疫反应和促进衰老凋亡等来发挥抑制肿瘤作用，达到肿瘤治疗目的。因此基于HDAC的治疗方法^[11]^受到广泛关注，是近年来较为有效肿瘤治疗策略。HDACi是一个大家族^[12]^，可依据不同结构分为四个不同的化学类别：①脂族酸，如丙戊酸和丁酸钠; ②环肽，如罗米地辛（Romidepsin）、曲帕辛（Trapoxin A）; ③羟基化合物，如曲古菌素A（Trichostatin A, TSA）和伏立诺他（Vorinostat, SAHA）; ④苯并酰胺类药物，如恩替诺特（Entinostat）和莫替诺司他（Mocetinostat）。值得一提的是，我国原创新药西达苯胺，在2015年全球获准上市用于抗肿瘤治疗，成为首个上市的亚型选择性口服HDACi。目前，四种HDACi：罗米地辛^[13]^、帕比司他^[14]^、贝利司他^[15]^和SAHA^[16]^获美国食品药品监督管理局（Food and Drug Administration, FDA）批准用于皮肤T细胞淋巴瘤（cutaneous T cell lymphoma, CTCL）、周围T细胞淋巴瘤（peripheral T cell lymphoma, PTCL）及多发性骨髓瘤（multiple myeloma, MM）的治疗。此外，许多其他类别HDACi正在各类癌症患者中开展临床试验。

## 2 HDACi和ICIs治疗

### 2.1 HDACi促进免疫正性调控

癌症发生发展过程中同时触发了宿主的固有免疫和适应性免疫。通常，肿瘤-免疫^[17]^是一个涉及肿瘤抗原释放呈递、效应T细胞（effector T cell, Teff）活化浸润、肿瘤细胞得以清除的循环过程。有效放大肿瘤抗原、抗原提呈机制（antigen presentation mechanism, APM）及Teff等在免疫反应中发挥的正向调节作用，有助于提高免疫抗肿瘤效应。

#### 2.1.1 HDACi诱导Teff浸润

Teff分泌多种免疫相关细胞因子，与自然杀伤细胞（natural killer cell, NK）构成机体抗肿瘤免疫的重要防线。研究^[18]^表明，CD8^+^肿瘤浸润T淋巴细胞（tumor infiltrating lymphocytes, TILs）耗竭引起的免疫逃逸是肿瘤抵制程序性死亡因子受体-1及配体（programmed cell death-1/programmed cell death ligand-1, PD-1/PD-L1）单抗的潜在机制，而使用HDACi可逆转这一现象。多数研究^[18-20]^发现，HDACi联合ICIs治疗，显著升高肿瘤微环境（tumor microenvironment, TME）中CD8^+^ T细胞和NK细胞水平，肿瘤体积明显缩小。小鼠肝癌模型中^[21]^，HDACi CG-745通过促进细胞毒性T淋巴细胞（cytotoxic T lymphocytes, CTL）和NK细胞的增殖放大PD-1单抗的抗肿瘤效应。机制研究^[20]^发现，HDACi可上调转录因子Bcl-6、Eomes、缺氧诱导因子-1（hypoxia-inducible factor-1, HIF-1）和T-bet表达，激活下游AKT-mTOR-p65通路，进而增强CTL的免疫效应。Christmas^[22]^发现HDACi联合抗PD-1或抗细胞毒性T淋巴细胞相关蛋白4（cytotoxic T-lymphocyte-associated protein 4, CTLA-4）抗体，促进活化的CD8^+^ T效应细胞产生颗粒酶b，显著改善了固有免疫细胞的浸润和功能，使适应性免疫反应更强。此外，伏立诺他联合派姆单抗治疗非小细胞肺癌的Ⅰ期临床试验^[23]^发现，患者获益程度与肿瘤间质内CD8^+^ T细胞水平相关，治疗后活检也证实，受益患者间质中这一细胞的含量升高，提示TILs在HDACi联合ICIs治疗中发挥重要作用。

#### 2.1.2 HDACi促进细胞因子释放

激活的免疫细胞通过释放大量细胞因子，结合相应受体参与免疫应答的各个环节。Cauwels等^[24]^观察到，把加工优化后具有靶向性的Ⅰ型干扰素（interferon, IFN）注入黑色素瘤小鼠体内，相比输注无靶向性IFN，肿瘤体积明显减小，且小鼠体重有所上升。研究^[25]^发现，HDACi通过靶向Ⅰ型IFN信号通路，降低体内卵巢上皮癌肿瘤负荷，延长生存率。多项研究^[26, 27]^显示，HDACi协同PD-L1或CTLA-4单抗通过诱导CD8^+^ T细胞生产肿瘤坏死因子（tumor necrosis factor, TNF）α、IFN-γ等细胞因子，进一步增强抗肿瘤免疫力。同时使用恩替诺特和PD-1单抗后^[6]^，体内细胞因子、趋化因子释放显著增多，有效抑制肿瘤生长。值得关注的是，在外周T细胞淋巴瘤的临床试验中^[28]^，体内PD-1（+）细胞居多的患者存在IFN-γ水平表达低下，细胞毒性反应受损等免疫缺陷，而西达苯胺可能恢复上述缺陷并上调适应性免疫相关的基因表达。所以说，HDACi有可能通过细胞因子触发的免疫级联反应放大ICIs的抗肿瘤效应。

#### 2.1.3 HDACi修复APM缺陷

越来越多的研究证据^[18, 29, 30]^表明，肿瘤抗原表达能力下降及APM受损是发生免疫逃逸的原因之一。Dibbern等^[31]^评估了宫颈鳞癌患者体内PD-L1和MHC Ⅰ类合并A、B、C重链的水平，发现在PD-L1（+）患者中，失去MHC Ⅰ类分子表达的占38%，PD-L1单抗治疗效果不佳，提示MHC Ⅰ类缺失导致APM受损，与针对PD-1/PD-L1轴的检查点抑制剂疗效有限相关。有学者^[29]^认为，莫替诺司他与IFN-γ协同上调人类白细胞抗原（human leukocyte antigen, *HLA*）基因的主要调控因子Ⅱ级反式激活因子（class Ⅱ trans-activator, CIITA），逆转了因抗原加工提呈减弱所引起的HLA-I表达下调，增加肿瘤免疫原性从而提高对PD-1单抗敏感性。另外，有研究^[32]^发现，晚期黑色素瘤患者接受2周的Domatinostat治疗后，体内APM及MHC Ⅰ、Ⅱ类分子的相关基因表达显著升高，且与PD-1/PD-L1阻断剂联合后抗肿瘤效果显著高于单药治疗。

#### 2.1.4 HDACi调控PD-L1表达

研究^[33]^显示，特异性阻断PD-L1会引起T细胞移动能力下降，因而免疫检查点治疗耐药的潜在机制包括PD-L1表达缺失。Iwasa等^[34]^研究发现，帕比司他通过激活转录启动子直接上调PD-L1的表达水平，同时也能够提高体内IFN-γ水平，介导STAT1-IRF1通路的激活间接上调PD-L1表达。有研究^[35]^显示，HDAC3i可快速增加*PD-L1*基因启动子区组蛋白乙酰化和溴域蛋白BRD4的募集，使转录激活; 此外，HDAC3i降低了DNMT1蛋白水平，间接激活PD-L1转录; 并且，CTLA-4和PD-1联合用药可导致早期肿瘤抗原提呈细胞上PD-L1的上调和肿瘤浸润效应T细胞上PD-1的表达。在小鼠黑色素瘤及乳腺癌肿瘤模型中^[36, 37]^，HDACi诱导PD-L1基因组蛋白迅速发生乙酰化，激发持久的基因表达，显著增强了体内PD-1/CTLA-4的阻断反应，与单药治疗相比，接受联合治疗的小鼠肿瘤进展缓慢，生存率更高。值得一提的是，Hu^[38]^却发现胰腺癌PD-L1高表达，使用HDAC3抑制剂RGFP966可通过干扰HDAC3-STAT3-PD-L1信号通路下调PD-L1的mRNA和蛋白水平，从而降低PD-L1单抗抗肿瘤疗效。可见，HDACi对不同肿瘤细胞PD-L1水平的调控有着双重效应，应多方面了解其作用机制选择适宜联合治疗方案。

### 2.2 HDACi抑制免疫负性调控

肿瘤抗免疫治疗的外在因素主要为免疫抑制细胞群的存在，如调节性T细胞（regulatory T cells, Tregs）、肿瘤相关巨噬细胞（tumor-associated macrophages, TAMs）和骨髓源性抑制细胞（myeloid-derived suppressor cells, MDSCs）。这些细胞产生并分泌免疫抑制因子，并表达与T细胞受体结合的抑制配体，营造适宜肿瘤生长的环境^[39]^。

#### 2.2.1 HDACi抑制Treg浸润

Treg依赖抑制Teff免疫应答以维持自身耐受性。研究^[40]^发现，Treg表面组成性高表达CTLA-4，与APC上的共抑制配体CD80/CD86结合后抑制Teff增殖活化，介导免疫逃逸。此外，越来越多的研究^[41, 42]^发现，大量的Treg细胞浸润到人和小鼠的各种肿瘤中; Briere等^[29]^体外试验发现，HDACi处理的Treg细胞转录因子FoxP3和HELIOS表达明显下调。重要的是，肿瘤浸润性CD8^+^ T细胞与FoxP3^+^ Treg细胞比率下降与不良预后相关。而在小鼠肝癌模型中^[27]^，联合使用HDACi和CTLA-4单抗后，使瘤内Treg细胞显著减少，产生强烈抗肿瘤效应。然而，全身性的Treg细胞消耗可能同时引发有害的自身免疫反应^[43]^。因此，HDACi如何在不引起自身免疫的情况下，减少瘤体内Treg浸润并激发有效的抗肿瘤免疫，值得进一步探究。

#### 2.2.2 HDACi调控TAMs表型

TAM通常分为肿瘤杀伤型（M1样细胞）和肿瘤支持型（M2样细胞）两类，但多表现为免疫抑制M2样表型。有关胰腺癌临床前试验^[19]^发现，经过伏立诺他联合索拉非尼预处理，肿瘤组织周围M1型TAM、CD8^+^ T细胞和NK细胞水平升高，在此基础上加入PD-1抑制剂，这些免疫细胞数量进一步增加，肿瘤生长得以有效抑制。Knox等^[44]^发现，黑色素瘤动物模型中，特异性HDAC6抑制剂与PD-1单抗的组合使得M2型TAM急剧减少，间接提高肿瘤浸润淋巴细胞水平，达到抑制肿瘤目的。还有研究^[21]^提示，在胰腺癌、结直肠癌和非小细胞肺癌中，HDACi抑制M2巨噬细胞极化，改变TME以增强PD-1单抗的抗癌作用。此外，有关结直肠癌研究报道^[45]^，与M2/M1 > 3的患者相比，M2/M1 < 3的患者的中位无进展生存期和总生存期分别延长至24.2个月、44.3个月，可见针对TAMs的抗肿瘤免疫治疗中，在抑制M2型TAM极化的同时也要积极促进向M1样表型的转化，而HDACi与PD-1/PD-L1的联合治疗恰好具有这一作用，但相关机制研究尚需进一步深入。

#### 2.2.3 HDACi介导MDSCs失能

MDSCs在募集Tregs的同时抑制CTLs、树突状细胞和NK细胞等，创造一个免疫抑制微环境，从数量或功能上介导MDSCs失能，可显著改善固有免疫细胞的浸润和功能。多项研究^[21, 22, 29]^证实，HDACi有效减少肿瘤微环境中MDSCs数量，解除对细胞因子、趋化因子释放的封锁，使得微环境由免疫抑制朝着肿瘤抑制方向转变。研究^[6]^发现，恩替诺特与PD-1抑制剂共同作用于肺癌、肾细胞癌动物模型后，体内肿瘤生长减缓、存活率提高，可能是由于恩替诺特诱发MDSCs功能改变相关，引起精氨酸酶1（arginase-1, ARG1）、诱导型一氧化氮合酶（inducible nitric oxide synthase, iNOS）和COX-2水平显著降低。类似的研究在黑色素瘤中也得到证实^[26]^，丙戊酸联合抗PD-1抗体的治疗，虽然增加了M-MDSCs数量的积累，但降低了M-MDSCs免疫抑制分子IL-10、IL-6和ARG1等的表达，增加了免疫刺激分子IL-12的表达，从而一定程度上抑制MDSCs功能发挥。

### 2.3 HDACi联合ICIs的临床研究

近年来，大量HDACi联合ICIs的临床试验正在各类癌症中开展（表 1）。其中，多数试验主要针对淋巴瘤、黑色素瘤等非实体瘤进行研究，而关于肺癌、胃肠道肿瘤等实体瘤的研究则较为少见。并且，临床试验中施加的治疗措施大多是HDACi和抗PD-1/PD-L1抗体的组合，HDACi联合其他类型ICIs的研究设计屈指可数。不过，随着深入研究HDACi及ICIs在抗肿瘤治疗中所发挥的作用，相信在不久的将来，HDACi与ICIs的联合会在更广泛的肿瘤研究领域中出现并最终用于临床抗肿瘤治疗。

**1:** HDACi联合ICIs的临床研究（数据来自ClinicalTrials.gov收集截止至2020.6.10） Clinical study of HDACi combined with ICIs (data collected by ClinicalTrials.gov as of June 10, 2020)

NCT	Study design	Cancer	Treatment	Outcome measures	Estimated enrollment	Recruitment status
03565406	Phase Ⅰb	Stage Ⅲ/Ⅳ melanoma	Mocetinostat induction phase+Ipilimumab+Nivolumab	DLTs, MTD	11	Terminated
03993626	Phase Ⅰb/ Ⅱ	Colorectal carcinoma	CXD101+Nivolumab	iDCR, iCR	55	Active, not recruiting
02393794	Phase Ⅰ/Ⅱ	Triple negative breast cancer	Romidepsin+Cisplatin+Nivolumab	Phase Ⅰ：Recommended phase Ⅱ dose of romidepsin in combination with cisplatin Phase Ⅱ：Objective response rate	54	Suspended
02453620	Phase Ⅰ	Breast carcinoma	Entinostat+Ipilimumab+Nivolumab	Incidence of adverse events	45	Active, not recruiting
02954991	Phase Ⅱ	Non-small cell lung cancer	Glesatinib+Nivolumab Sitravatinib+Nivolumab Mocetinostat+Nivolumab	Number of patients experiencing tumor size reduction	280	Active, not recruiting
02935790	Phase Ⅰ	Malignant melanoma	ACY-241+Ipilimumab+Nivolumab	MTD	1	Completed
03838042	Phase Ⅰ/Ⅱ	Children and adolescents with high-risk refractory malignancies	Nivolumab+Entinostat	StageⅠ：DLTs; StageⅡ：CR, PR	128	Recruiting
02635061	Phase Ⅰ	Non-small cell lung cancer	ACY-241+Nivolumab	Incidence of adverse events, recommended Phase 2 dose	16	Active, not recruiting
03552380	Phase Ⅱ	Renal cell carcinoma	Entinostat+Entinostat+Ipilimumab	QRR, recommended Phase Ⅱ dose	53	Active, not recruiting
03250273	Phase Ⅱ	Cholangiocarcinoma, pancreatic cancer	Entinostat+Nivolumab	QRR	54	Active, not recruiting
04315155	Phase Ⅰ	Metastatic adenocarcinoma	Belinostat+Nivolumab+Ipilimumab	DLTs	39	Not yet recruiting
01928576	Phase Ⅱ	Non-small cell lung cancer	Entinostat+Nivolumab+Azacitidine	OR	120	Recruiting
03873025	Phase Ⅰ/Ⅱ	Diffuse large B-cell lymphoma	CXD101+Pembrolizumab	MTD, BOR	45	Not yet recruiting
02437136	Phase Ⅰ/Ⅱ	Non-small cell lung cancer, melanoma, colorectal cancer	Entinostat+Pembrolizumab	Incidence of adverse events	202	Unknown
03426891	Phase Ⅰ	Glioblastoma	Vorinostat+Pembrolizumab+Temozolomide	MTD	32	Recruiting
02909452	Phase Ⅰ	Solid tumors	Entinostat+Pembrolizumab	Incidence of treatment-emergent adverse events	30	Unknown
03765229	Phase Ⅰ/Ⅱ	Melanoma	Entinostat+Pembrolizumab	Incidence of conversion of non-inflamed to inflamed melanoma	14	Recruiting
03978624	Phase Ⅰ/Ⅱ	Bladder cancer	Entinostat+Pembrolizumab	Change from baseline in Z-score of T cell CD8 immune 37-gene signature	20	Recruiting
04357873	Phase Ⅱ	Squamous cell carcinoma	Vorinostat+Pembrolizumab	ORR	111	Recruiting
03179930	Phase Ⅱ	Lymphomas	Entinostat+Pembrolizumab	Response	47	Recruiting
03220477	Phase Ⅰ	Lung cancer	Mocetinostat+Guadecitabine+Pembrolizumab	RR	40	Recruiting
03150329	Phase Ⅰ	Lymphoma	Vorinostat+Pembrolizumab	Incidence of adverse events	60	Recruiting
03590054	Phase Ⅰ	Melanoma	Abexinostat+Pembrolizumab	MTD, ORR	40	Recruiting
NCT	Study design	Cancer	Treatment	Outcome Measures	Estimated enrollment	Recruitment status
02619253	Phase Ⅰ	Renal cell carcinoma, urinary bladder neoplasms	Abexinostat+Pembrolizumab	Recommended phase 2 dose	57	Active, not recruiting
02538510	Phase Ⅰ/Ⅱ	Head and neck squamous cell carcinoma	Vorinostat+Pembrolizumab	Incidence of toxicity graded	49	Active, not recruiting
02697630	Phase Ⅱ	Metastatic uveal melanoma	Entinostat+Pembrolizumab	ORR	29	Active, not recruiting
02638090	Phase Ⅱ	Lung cancer	Vorinostat+Pembrolizumab	MTD	100	Recruiting
04190056	Phase Ⅱ	Breast cancer	Vorinostat+Tamoxifen+Pembrolizumab	ORR	65	Not yet recruiting
02395627	Phase Ⅱ	Breast neoplasms	Vorinostat+Tamoxifen+Pembrolizumab	ORR, treatment-related adverse events	38	Active, not recruiting
03820596	Phase Ⅰ/Ⅱ	Extranodal natural killer/T cell lymphoma	ChidamideSintilimab	Phase Ⅰ: DLTs, MTD, Maximum tolerable dose Phase Ⅱ：PFS, DOR, OS	50	Recruiting
02708680	Phase Ⅰ/Ⅱ	Breast cancer	Entinostat+Atezolizumab	DLTs, MTD, ORR, OS	88	Unknown
03024437	Phase Ⅰ/Ⅱ	Renal cancer	Entinostat+Bevacizumab+Atezolizumab	Recommended phase Ⅱ dose，ORR	62	Recruiting
03280563	Phase Ⅰ/Ⅱ	Breast neoplasms	Entinostat+Atezolizumab+Fulvestrant	ORR	126	Recruiting
02805660	Phase Ⅰ/Ⅱ	Advanced cancer	Mocetinostat+Durvalumab	DLTs	119	Active, not recruiting
02915523	Phase Ⅰ/Ⅱ	Epithelial ovarian cancer, peritoneal cancer, Fallopian tube cancer	Entinostat+Avelumab	Recommended phase 2 dose, PFS, ORR, OS	140	Active, not recruiting
02032810	Phase Ⅰ/Ⅱ	Melanoma, skin cancer	Panobinostat+Ipilimumab	ORR, PFS, OS	17	Active, not recruiting
04296942	Phase Ⅰ	Breast cancer	Entinostat+M7824+BN-Brachyury+T-DM1	ORR, PFS	55	Not yet recruiting
04040491	Single-arm, Multi-center Clinical study	Peripheral T-cell lymphoma	Chidamide+PD-1 blocking antibody+Lenalidomide+gemcitabine	ORR, PFS, OS	100	Recruiting
04038411	Single-arm, multi-center clinical study	NK/T cell lymphoma	Chidamide+PD-1 blocking antibody+Lenalidomide + Etoposide phosphate	ORR, PFS, OS	50	Recruiting
04337606	Open-label Phase Ⅰ/Ⅱ trial	Non Hodgkin lymphoma	Chidamide+Camrelizumab+Decitabine	CRR, Adverse events	100	Recruiting
04512534	Phase Ⅱ	Peripheral T-cell lymphoma	Chidamide+Sintilimab	PFS, OS	51	Recruiting
04025931	Phase Ⅱ	Sarcoma	Chidamide+Toripalimab	ORR, PFS, OS, DCR	53	Recruiting
04414969	Open-label, multicenter, phase Ⅱ	NK/T cell lymphoma	Chidamide+Anti-PD-1 antibody+Peg-Asparaginase	ORR, PFS, OS, CRR	35	Recruiting
MTD: Maximum tolerance dose; DLTs: dose limiting toxicity; iDCR: immune disease control rate; iCR: immune complete response; iSD: immune stable disease; PR: partial response; ORR: objective response rate; BOR: best objective response rate; PFS: progression free survival; DOR: duration of response; CRR: complete response rate; RR: response rate.						

## 3 小结和展望

ICIs是近年来研究的热点，已成为晚期癌症患者的主要治疗选择之一。遗憾的是，免疫耐药的发生令ICIs的疗效大打折扣，而HDACi联合ICIs治疗为解决这一问题带来了新的研究思路，HDACi通过调控免疫反应的正/负向调节机制，给ICIs疗效的发挥提供了双重保障，但当前相关的实验和研究报道十分有限，尚停留在PD-1/PD-L1及CTLA-4单抗与HDACi联合抗肿瘤治疗及相关表浅机制的探讨。因此，多类型HDACi协同ICIs治疗及深入作用机制的研究还有待更进一步探索。HDACi联合ICIs在恶性肿瘤Ⅰ期/Ⅱ期临床试验中初见成效，显著延长肿瘤患者无病生存期，可见，深入探究二者协同抗肿瘤机制并合理设计用药方案具有可观的研究前景，使免疫治疗更好造福于癌症患者。
